# The Additive Manufacturing of Aluminum Matrix Nano Al_2_O_3_ Composites Produced via Friction Stir Deposition Using Different Initial Material Conditions

**DOI:** 10.3390/ma15082926

**Published:** 2022-04-17

**Authors:** Mohamed M. El-Sayed Seleman, Sabbah Ataya, Mohamed M. Z. Ahmed, Ahmed M. M. Hassan, Fahamsyah H. Latief, Khalil Hajlaoui, Ahmed E. El-Nikhaily, Mohamed I. A. Habba

**Affiliations:** 1 Department of Metallurgical and Materials Engineering, Faculty of Petroleum and Mining Engineering, Suez University, Suez 43512, Egypt; mohamed.elnagar@suezuniv.edu.eg; 2 Department of Mechanical Engineering, College of Engineering, Imam Mohammad Ibn Saud Islamic University, Riyadh 11432, Saudi Arabia; smataya@imamu.edu.sa (S.A.); fhlatief@imamu.edu.sa (F.H.L.); kmhajlaoui@imamu.edu.sa (K.H.); 3 Mechanical Engineering Department, College of Engineering at Al Kharj, Prince Sattam Bin Abdulaziz University, Al Kharj 16273, Saudi Arabia; 4 Mechanical Department, Faculty of Technology and Education, Suez University, Suez 43518, Egypt; ahmed.mostafa@ind.suezuni.edu.eg (A.M.M.H.); ahmed.eassa@ind.suezuni.edu.eg (A.E.E.-N.); mohamed.atia@suezuniv.edu.eg (M.I.A.H.)

**Keywords:** additive manufacturing, friction stir deposition, AA2011, nanocomposites, temper conditions, hardness, compressive strength, wear resistance

## Abstract

The current work investigates the viability of utilizing a friction stir deposition (FSD) technique to fabricate continuous multilayer high-performance, metal-based nanoceramic composites. For this purpose, AA2011/nano Al_2_O_3_ composites were successfully produced using AA2011 as a matrix in two temper conditions (i.e., AA2011-T6 and AA2011-O). The deposition of matrices without nano Al_2_O_3_ addition was also friction stir deposited for comparison purposes. The deposition process parameters were an 800 rpm rod rotation speed and a 5 mm/min feed rate. Relative density and mechanical properties (i.e., hardness, compressive strength, and wear resistance) were evaluated on the base materials, deposited matrices, and produced composites. The microstructural features of the base materials and the friction stir deposited materials were investigated using an optical microscope (OM) and a scanning electron microscope (SEM) equipped with an EDS analysis system. The worn surface was also examined using SEM. The suggested technique with the applied parameters succeeded in producing defect-free deposited continuous multilayer AA2011-T6/nano Al_2_O_3_ and AA2011-O/nano Al_2_O_3_ composites, revealing well-bonded layers, grain refined microstructures, and homogeneously distributed Al_2_O_3_ particles. The deposited composites showed higher hardness, compressive strengths, and wear resistance than the deposited AA2011 matrices at the two temper conditions. Using the AA2011-T6 temper condition as a matrix, the produced composite showed the highest wear resistance among all the deposited and base materials.

## 1. Introduction

Aluminum matrix ceramic composites (Al-MCCs) are of strong interest in the design of engineering parts in a vast number of industrial applications [1,2,3]. There are different uses for Al-MCCs in many sectors such as the aerospace, transportation, and marine industries. In general, Al-MCCs have superior properties, such as strength, hardness, and wear resistance, compared with aluminum alloys [4,5,6,7]. Different techniques have been used to introduce ceramic particles into aluminum matrix alloys: powder metallurgy (PM) [8,9,10], casting [11,12,13,14], fusion-based additive manufacturing (FB-AM) [15,16,17], and solid-state-based additive manufacturing (SS-AM) [18,19,20]. Producing complex 3D geometries of Al-MCCs using PM and casting techniques is difficult. Moreover, applying the FB-AM technique to produce Al-MCCs is a challenge due to the weak wettability between the liquid matrix and reinforcement phases [21] and, in some cases, the chemical reactions between the two phases result in undesired compounds [22]. Furthermore, using the FB-AM technique, the produced additive manufacturing composite parts have several flaws, especially for Al-MCCs, such as quick solidifications, hot cracking, pores, and agglomeration of dispersed oxide particles [23,24]. The limitations mentioned above have been necessary to discover and try alternative technologies to develop Al-MCCs. Nowadays, friction additive manufacturing (FAM) technology is an effective technology to outdo the limitations of FB-AM and successfully produce Al-based composites for many industrial applications, e.g., biomedical, aerospace, marine, and transportation [25,26]. This technology is suitable for various materials, such as ceramics, metals, and composites, with more complex geometric designs, mass customization, and much less material waste [23,27]. The FAM technology can be classified into friction stir additive manufacturing, friction surfacing additive manufacturing (using the hollow shoulder or consumable rod), and friction stir deposition (FSD) [21,23,28]. In friction stir additive manufacturing, the plates are joined one over the other using a friction stir welding tool. The tool pin length is longer than the thickness of the one plate to ensure the joining of the two plates at one time [29,30,31]. The effect of the addition of SiC on the six layers of AA5059-O produced using friction stir additive manufacturing with a threaded tapered pin at 63 mm/min, 450 rpm, and a 2° tilt angle was investigated by Srivastava and Rathee [32]. The results revealed good bonding between the SiC particle and the AA5059-O, and the hardness of the composite achieved 140 HV compared with 85 HV for the base material. Tan et al. [19] successfully produced four AA6061-T6/nano Al_2_O_3_ composite layers via friction stir additive manufacturing at 1000 rpm and 100 mm/min using different Al_2_O_3_ sizes. They concluded that the produced AA6061-T6/nano Al_2_O_3_ composites had higher hardness compared to the additive materials without Al_2_O_3_ addition. Friction surfacing additive manufacturing depends on friction stir principles and enables the production of surface composite coatings via two techniques. The first one utilizes a consumable rod filled with ceramic particles. The second one inserts a mixture of ceramic particles and metal matrix inside a hollow tool shoulder and deposits the mixture on the surface to achieve surface composite coatings. Gandra et al. [18] produced three layers 20 mm in diameter of AA6082/SiC coating surface composite on an AA2024-T3 substrate plate using the consumable rod technique filled with different microsizes of SiC. The authors concluded that the hardness of the deposited composite layers was higher than the deposited layers without SiC. In addition, the addition of SiC particles enhanced the wear performance of the AA6082-T6 layers. The FSD process (a solid-state process) can be applied with a wide range of metals and alloys [33,34,35] to produce continuous multilayer additive manufacturing parts (AMPs) [36,37] and additive manufacturing composite parts (AMCPs) [20,38]. This technique promotes continuous feeding of a rotating consumable rod towards a fixed substrate plate through rubbing action (stirring process). This high friction generates torsional shear stress between the plasticized material and rotating rod, producing deposited continuous layers on the substrate [39,40,41,42,43]. Karthik et al. [38] utilized this technique to produce an AA5083/ 6 Vol% titanium particle metal–metal composite. The results showed the composite had much better compressive strength, and the microstructure of the deposited material showed uniformly distributed Ti particles. Moreover, Karthik et al. [44] successfully produced another system of metal–metal composite (i.e., AA5083/CoCrFeNi high-entropy alloy particles) using the same technique. They concluded that the compressive strength and the bulk hardness of the composite were more than 1.8 times higher than that of the AA5083-H112 base material. Dilip et al. [42] fabricated the AMPs using FSD with 20 mm diameter AA2014-T6 rods. The produced AMPs consisted of five well-bonded layers with microstructures with fine grains and refined second-phase particles. Alzahrani et al. [45] succeeded in depositing an A356 cast alloy using the FSD process at different feeding rates. Compared to the as-cast material, the grain size of the deposited material decreased by 97.9%, 95.0%, and 92.2% at feeding rates of 3, 4, and 5 mm/min, respectively, and the enhancement in hardness attained was 43.6%, 34.3%, and 29.7%, respectively. Ahmed et al. [46] studied the effect of temper condition and feeding speed rate on the fabrication of AA2011 AMPs utilizing the FSD process at a 1200 rpm rotation rod speed and 3, 6, and 9 mm/min feeding rates. According to microstructural analyses, significant grain refining and fine intermetallic particle were detected in the AMPs from the two temper conditions of AA2011-T6 and AA2011-O. According to the available literature review, there has been no attempt to disperse nanoceramic particles in high-strength aluminum alloys (2xxx series) via the FSD technique. In the present study, the FSD process was utilized to produce AA2011 nano Al_2_O_3_ composites. Two matrices of AA2011 (i.e., sAA2011-T6 and AA2011-O) were used. The deposited composite materials were evaluated in terms of physical and mechanical properties and compared with the deposited materials without nano Al_2_O_3_.

## 2. Materials and Methods

### 2.1. Initial Materials

In order to study the influence of the initial matrix conditions on the properties of the produced composites, two temper conditions (i.e., T6 and O) were utilized. The parent material was AA2011-T6 rods with a diameter of 40 mm and a length of 150 mm. The annealing procedure was carried out at 415 °C for 2.5 h, followed by slow cooling in the furnace to room temperature [46]. Table 1 shows the chemical composition of the as-received AA2011 material.

**Figure 1:**
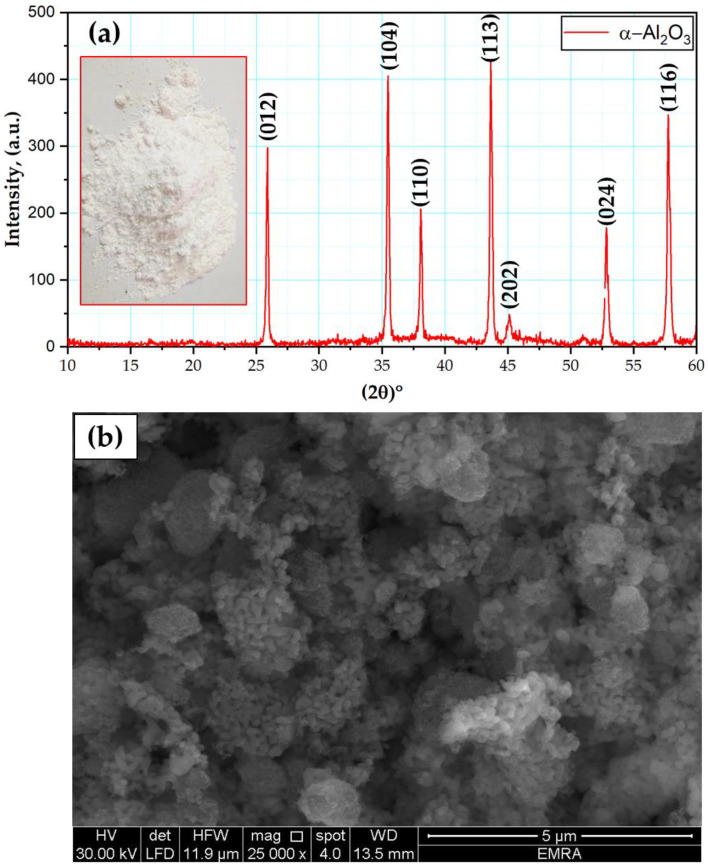
(**a**) XRD analysis and (**b**) SEM image of the Al_2_O_3_ powder.

Nano Al_2_O_3_ powder was supplied by Sigma-Aldrich (Burlington, MA, USA), and according to the supplier, the purity was 99.99%. Figure 1 shows the XRD analysis (Figure 1a) and an SEM micrograph (Figure 1b) of the morphologies of the Al_2_O_3_ particles. The XRD patterns confirm the high purity of the Al_2_O_3_ powder, and the SEM image shows that the nano Al_2_O_3_ powder was irregular in shape with a particle size ranging from 20 to 40 nm.

**Figure 2:**
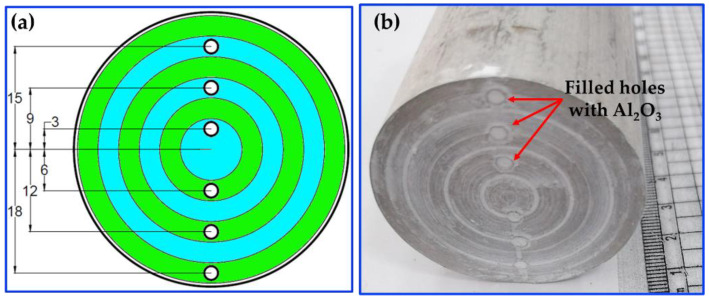
(**a**) Design sketch of the machined holes; (**b**) the holes filled with Al_2_O_3_.

A spark electric discharge machining (SK703-3040 CNC EDM drilling machine, Xiang Cheng, Suzhou, China) was used to drill holes for the AA2011-T6 and AA2011-O consumable rods with a diameter of 2 mm and a depth of 50 mm; the number of holes was six. The design of the distribution of the holes is shown in Figure 2a. Before the FSD process, the six drilled holes were filled with the nano Al_2_O_3_ powder (Figure 2b).

### 2.2. Production of Additive Manufacturing Composites

**Figure 3:**
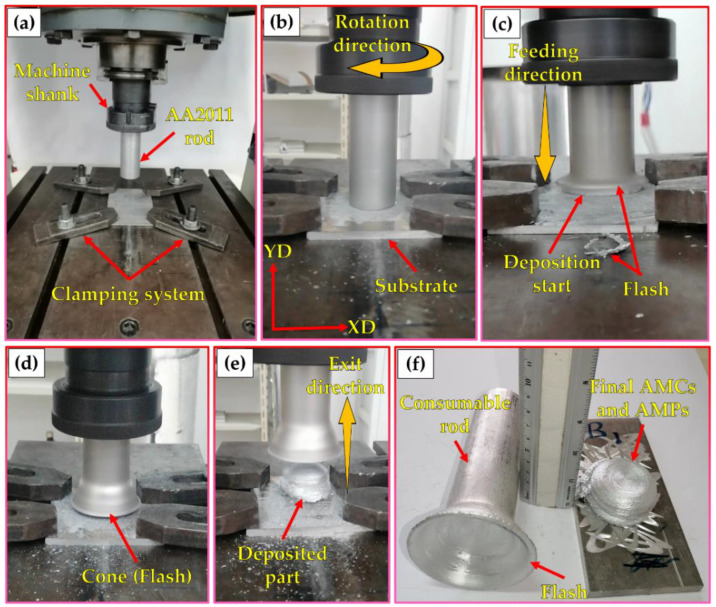
Photographs of the FSD process of AA2011: (**a**) setup of FSD and the consumable rod and substrate ready for the process; (**b**) start of the FSD; (**c**) start of the deposition; (**d**) formation of a cone flash around the deposits; (**e**) end of the deposition process; (**f**) the final deposited material and the rest of the consumable rod.

A friction stir welding/processing machine (EG-FSW-M1, Suez University, Suez, Egypt) was used to produce the additive manufacturing composites (AMCs) via the FSD technique [43]. Figure 3 shows pictures of the FSD process applied to produce AMCs. The FSD process involved three steps: (1) fixing the consumable AA2011 rods in the machine shank to ensure the complete fixation of the rods throughout the FSD process (Figure 3a); (2) rotating it at a constant rotation speed (800 rpm) while moving downward to reach the AA6082 substrate plate (Figure 3b); (3) finally, under a continuous feeding speed (5 mm/min) for the deposition of the two temper conditions, AA2011-T6 and AA2011-O (Figure 3c), the rods are plastically deformed due to the generated frictional heat between the rotating rods and the substrate plate that caused the material to deposit from the rotation rods to the substrate plate to build continuous layers of AMCs (Figure 3d,e). The appearance of the consumed rod end tends to form a conical shape with a thin section as shown in Figure 3f. Additive manufacturing parts (AMPs) from AA2011-T6 and AA2011-O were also friction stir deposited (FSDed) without Al_2_O_3_ addition using the same processing parameters for comparison purposes to investigate the role of the nano Al_2_O_3_ reinforcement on the behavior and properties of the FSDed composites.

### 2.3. Characterization of AMPs and AMCs

**Figure 4:**
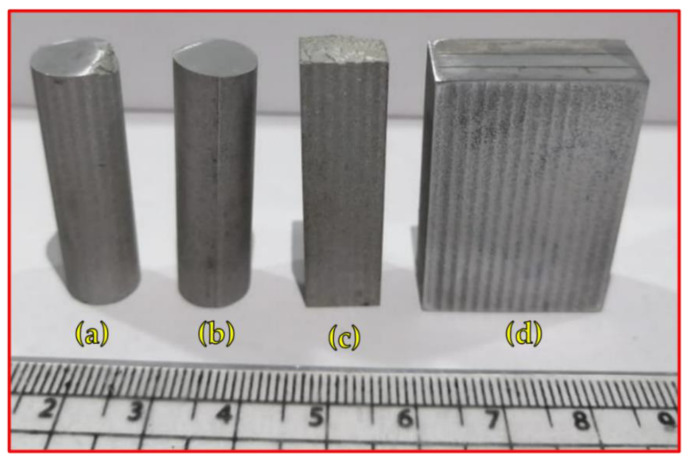
Image of the cut specimens from produced AMPs and AMCs for further investigation: (**a**) compression test sample; (**b**) wear test sample; (**c**) density measurement sample; (**d**) hardness and microstructure sample.

For each building condition of the fabricated AMPs and AMCs, four test specimens were cut vertically (parallel to the build direction, YD axis) by a wire cut machine (DK77 High-Speed EDM wire cutting machine, Jiangsu, China), as shown in Figure 4, to be used in the characterization processes. Table 2 illustrates the specification and objectives of each test specimen. The cut specimens of the AA2011 initial conditions, AMPs, and AMCs were investigated in terms of microstructure, physical properties, and mechanical properties (i.e., hardness and compression). Furthermore, the wear behavior was also evaluated.

**Figure 5:**
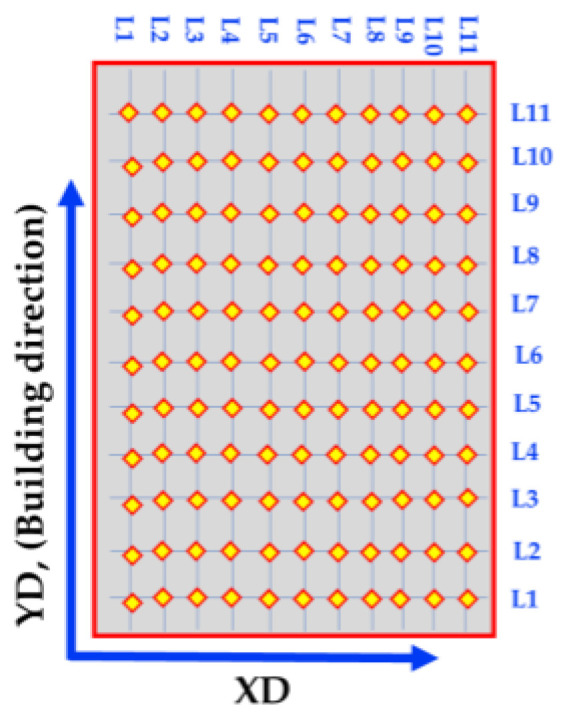
Sketch of hardness map measurements of AMPs and AMCs.

The OM examination was carried out using an optical microscope (Olympus, BX41M-LED, Tokyo, Japan). The specimens were ground and polished to a 0.05 µm Al_2_O_3_ surface finish followed by chemical etching with Keller’s regent solution (i.e., 95 mL water, 2.5 mL HNO3, 1.5 mL HCL, and 1.0 HF), immersing for up to 20 s. Density was measured based on Archimedes’ principle using distilled water as a liquid medium according to the JIS R2205-1992 standard. Vickers hardness measurements were carried out using a Vickers hardness tester (Model: HWDV-75, TTS Unlimited Osaka, Japan) with a 0.2 Kg load and 15 s dwell time. The hardness maps were plotted by collecting eleven vertical and eleven horizontal lines with a 2 mm step as shown in Figure 5. The compression test was carried out using a universal testing machine (Model: WDW-300D Testing Machine, 30 tons, Guangdong, China) at a constant cross-head speed displacement rate of 1 mm/min at room temperature. The compression test specimen was 10 mm in diameter and 15 mm in height. Wear tests using a pin-on-disc technique were also conducted. The wear test samples were cylindrical with a 10 mm diameter and an effective height of 12 mm. The disc (counter surface) was a hardened steel disc (64 HRC) with a diameter of 110 mm. The tests were conducted for a 2072 m sliding distance at different loads of 10, 20, and 30 N. After the wear test, the final weight of the test specimen was recorded, and the wear rate was estimated using the weight loss. The SEM, equipped with an EDS analysis system (Model: QUANTA FEG 250, FEI company, Hillsboro, OR, USA), was used to characterize the microstructure of the AA2011-T6/nano Al_2_O_3_ composite and the worn surface specimens of the AA2011’s initial conditions, AMPs, and AMCs.

## 3. Results and Discussion

### 3.1. Fabricated AMPs and AMCs

**Figure 6:**
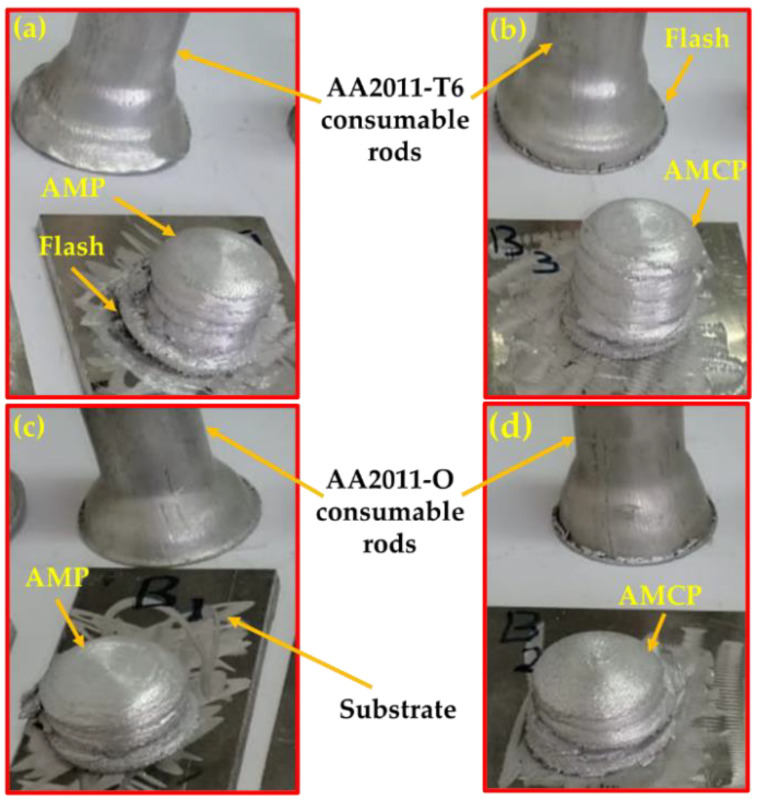
Photographs of the additive manufacturing materials: (**a**) AMP and (**b**) AMC using AA2011-T6 and (**c**) AMP and (**d**) AMC using AA2011-O deposited at an 800 rpm rotation speed and a 5 mm/min feed rate.

To carry out the FSD process and produce the AMPs or AMCs for the two temper conditions, AA2011-T6 and AA2011-O, with a diameter of 40 mm, several experiments were performed to observe the behavior of the consumable rod to avoid buckling of the rotating rod due to the FSD process and ensure the continuous building of layers of the AMPs or AMCs without any visual defects. Based on these experiments, a feed rate of 5 mm/min and a rotation speed of 800 rpm were considered as the processing parameters for fabricating AMCs in the current study. Figure 6 shows the deposited materials and the remains of the AA2011-T6 and AA2011-O consumable rods. The FSD process using a rotation speed of 800 rpm and a 5 mm/min feeding rate succeeded in fabricating the AMPs and AMCs at the two temper conditions of the initial consumable rod: AA2011-T6 (Figure 6a,b) and AA2011-O (Figure 6c,d).

**Figure 7:**
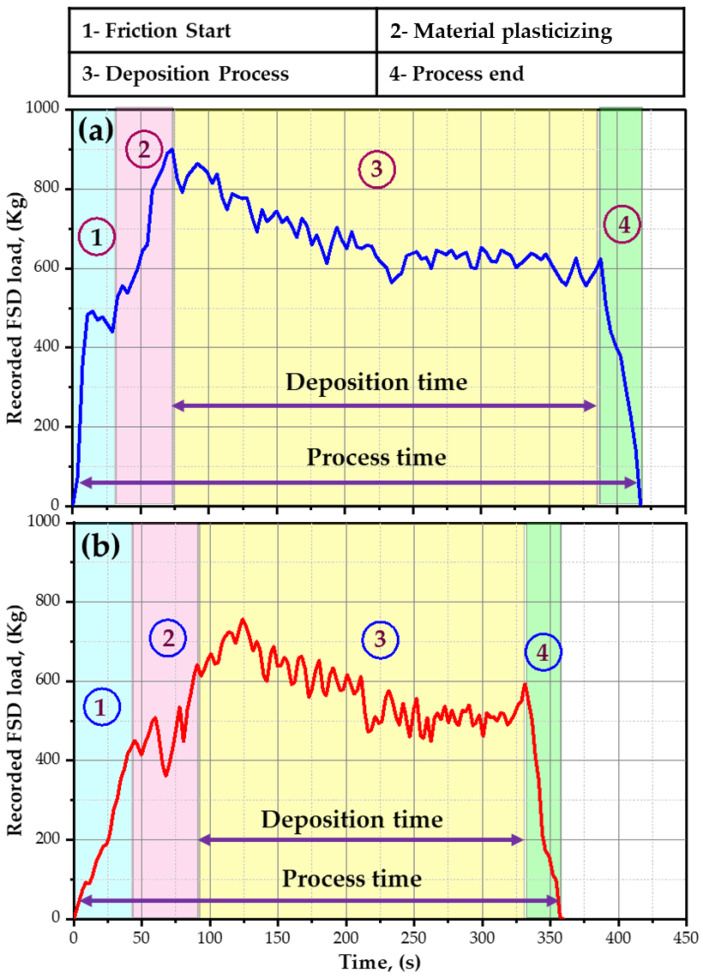
Illustrates the different stages and the recorded machine load during the FSD process to produce the AA2011/nano Al_2_O_3_ composites with the (**a**) AA2011-T6 matrix and (**b**) AA2011-O matrix.

According to the available data from the literature review, no research works clarify the deposition stages in producing Al-based composites via FSD. Moreover, the deposition load value given in the monitor for the FSW/FSP machine in the present work can be used as an indicator for the resistance of consumable rod material to deposit on the substrate plate, as it depends on the as-received material used. The FSD stages and the recorded load during the FSD process of the AA2011/nano Al_2_O_3_ composites using the two matrices, AA2011-T6 and AA2011-O, are given in Figure 7a,b, respectively. The FSD of the composite materials can be distinguished into four stages: friction onset, material plasticizing, deposition process, and process end. In the first stage, the rotating consumable rod touched the substrate surface, and then the recorded load suddenly increased due to the mutual friction between the rotating AA2011 consumable rod and the fixed substrate to achieve the friction onset. During the second stage, the stirring action between the consumable rod and the substrate with a continuous feeding rate resulted in sufficient frictional heat able to plasticize the material to form the first deposition layer. This stage could be recognized as the transition stage of the FSD process due to the instability of the recorded load as given in Figure 7. The third stage represents the building of composite materials in continuous layers, from bottom to top, at a nearly stable recorded load, for a particular period of time, called the deposition time. The initial material properties of the consumable rods controlled their behavior during the FSD, and this was related to the hardness of the materials. It was noted that there was a strong resistance to friction agitation for the hard material of the AA2011-T6 matrix than for the soft material of the AA2011-O during FSD when the other processing parameters (i.e., feeding rate, rotation speed, and consumable rod volume) were constant. Thus, the time needed to build continuous layers of AA2011-T6 was longer than that consumed in building AA1011-O, as given in Figure 7a,b, respectively. Finally, the deposition load decreased sharply at the end of the process because the rotating rod leaves upward after finishing the deposition process.

**Figure 8:**
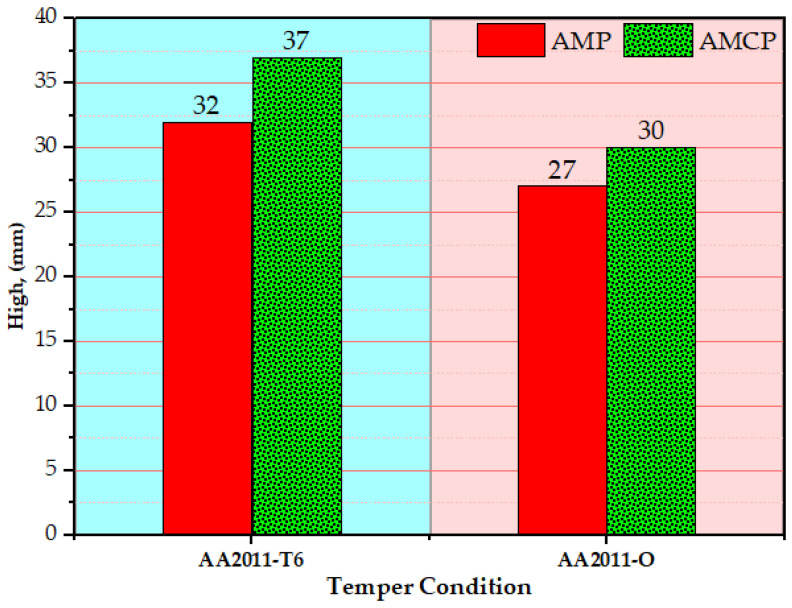
The height of AMPs and AMCPs against the temper conditions of AA2011-T6 and AA201-O.

Figure 8 shows the height of AMPs and AMCPs as a function of the temper condition of the AA2011 initial material. From the experimental results, it can be said that the height of the friction stir deposited material depended on the deposition time. The deposition time was related to the hardness of the consumable rod at a constant feeding rate [43,45,47]. Thus, the AA2011-T6 showed a higher multilayer building with a shorter diameter than the produced multilayer using the AA2011-O consumable rod, as a certain volume of the consumable rod was targeted to friction stir deposit as shown in Figure 8. Furthermore, the presence of the Al_2_O_3_ nanoceramic phase during the stirring process increased the resistance of the plasticized material to compatibility with an increase in the feeding rate, resulting in a higher building layer compared to that produced without alumina addition for the two temper AA2011 materials. This increase in height was at the expense of the increase in diameter.

### 3.2. Macrostructure and Microstructure Investigation

**Figure 9:**
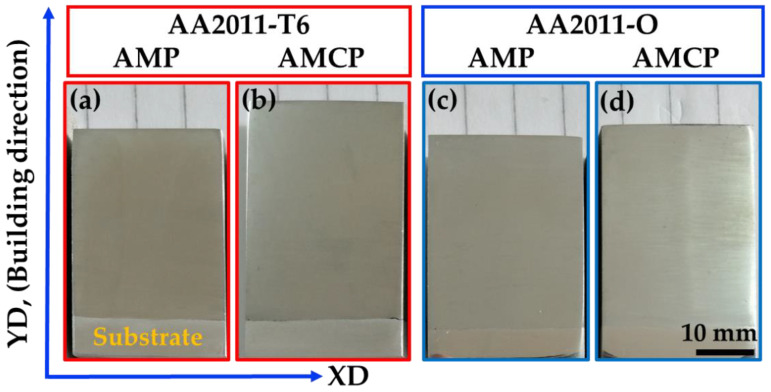
The macrostructures of the (**a**) AA2011-T6 AMP; (**b**) AA2011-T6 AMCP; (**c**) AA2011-O AMP; (**d**) AA2011-O AMCP produced at a rotation speed of 800 rpm and a 5 mm/min feed rate.

Figure 9 shows the macrostructure of the AMPs and AMCPs produced using a rotation speed of 800 rpm and a 5 mm/min feed rate at the two temper conditions of AA2011-T6 and AA2011-O. It should be noted that, as illustrated in Figure 9, the macrostructure of the deposited AMPs and AMCPs cross-sections demonstrated fully continuous structures without any bonding voids and defects.

**Figure 10:**
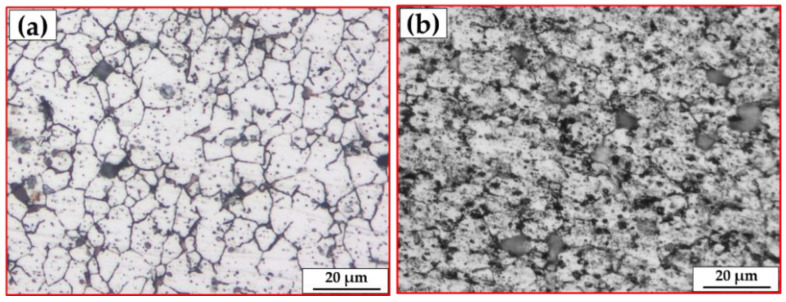
The OM microstructures of the initial material conditions of (**a**) AA2011-T6 and (**b**) AA2011-O.

**Figure 11:**
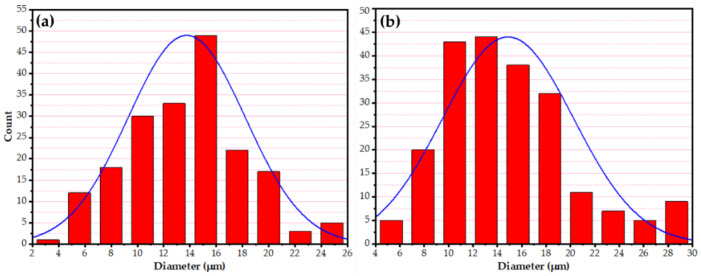
The grain size analysis of the initial material conditions of (**a**) AA2011-T6 and (**b**) AA2011-O.

The AA2011 BM had a high material hardness as a result of work hardening. The annealing temperature will lower the dislocation density after the annealing process. In addition, according to the Hall–Patch relationship, the grains continued to merge into larger grain sizes, resulting in a continuing decline in hardness and material softening properties [48,49]. Figure 10 depicts the microstructure of the initial consumable rod materials: (a) AA2011-T6 and (b) AA2011-O. The grain size analysis of the initial materials (i.e., AA2011-T6 and AA2011-O) (Figure 11) shows grain sizes from 8.62 to 25.32 µm with an average of 13.75 µm for the AA2011-T6 rod material. The grain size analysis of the AA2011-O shows a larger grain size than for the T6 temper condition; the grain size of the AA2011-O ranged from 5.29 to 29.46 µm with an average grain size of 14.87 µm.

**Figure 12:**
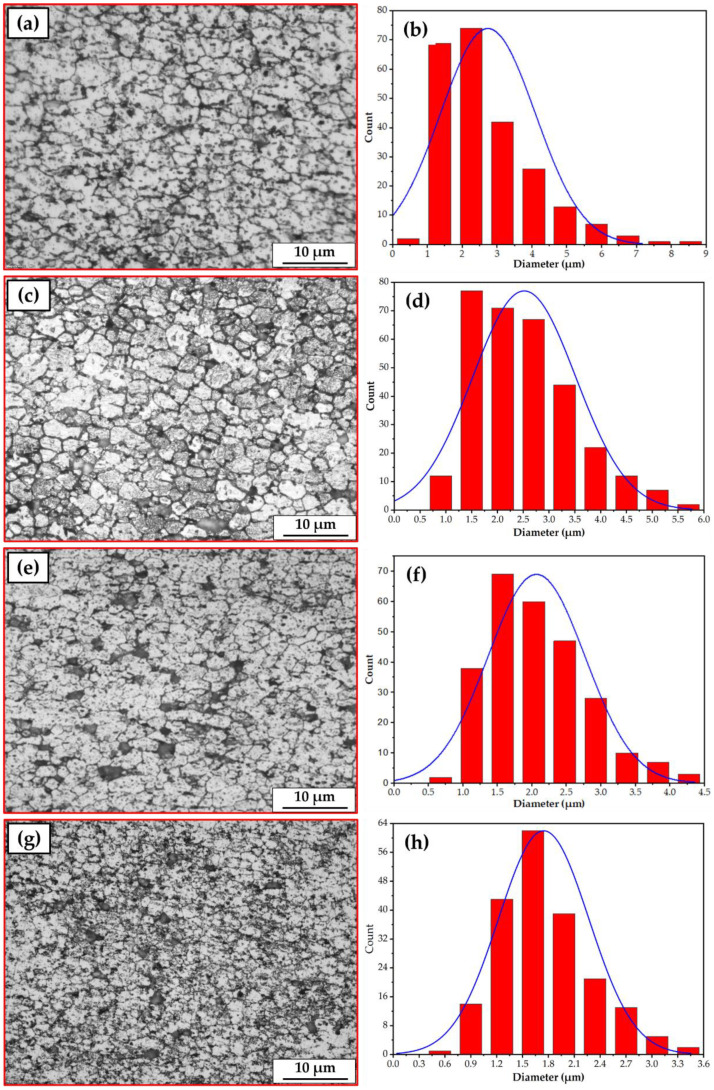
The microstructures of the deposited (**a**) AA2011-T6 AMP; (**b**) AA2011-O AMP; (**c**) AA2011-T6/Al_2_O_3_ composite; (**d**) AA2011-O/Al_2_O_3_ composite produced at a rotation speed of 800 rpm and a 5 mm/min feeding rate. The grain size analysis of the produced AMPs of (**e**) AA2011-T6 and (**f**) AA2011-O. (**g**) The AMCPs using the temper condition AA2011-T6 and (**h**) the AMCPs using the temper condition AA2011-O.

The FSD, as a thermomechanical process, is identical to friction stir processing (FSP) [45,46,50] in terms of heat mechanisms (i.e., generation, dissipation, and transfer) in the stir zone. In the continuous deposition process of AMPs and AMCPs, the frictional heat was generated between the rotating AA2011 (T6 and O temper conditions) consumable rods and the substrate plate during the dynamic contact friction process (DCF). Then, it caused severe plastic deformation (SPD) of the consumable rod material during the feeding rate and transferred the plasticized material to build AMP and AMCP during the deposition process. The deposited material’s microstructural features were nearly similar to those gained in the stir zone material (SZ) during the FSP [51,52]. During the FSD process, the material of the rotating consumable rod is subjected to SPD at high homologous temperatures. As a result, the friction deposited material undergoes dynamic recrystallization [46] and develops a very fine grain size. The OM observations of the microstructure indicated the presence of an equiaxed refined grain for the deposited AMPs and AMCPs, as shown in Figure 12a–d, compared to that shown in the initial materials (Figure 11). The FSD process produced significant grain refining with average grain sizes of 2.57 and 2.41 µm for the AA2011-T6 (Figure 12e) and AA2011-O (Figure 12f) deposited matrix materials. The as-received, extruded consumable rods, AA2011-T6 and AA2011-O, were altered from coarse grains to refined, equiaxed grains throughout the deposited microstructure of AMPs. These grain sizes were similar to those seen in FSW weld nuggets. The observed equiaxed grain refining was as severe as that occurring in the FSD of aluminum, which reported ultrafine grains [53,54,55]. This difference can be credited to the higher stacking fault energies inherent in aluminum alloys and is akin to other severe plastic deformation processes such as FSW and high-pressure torsion [34,42]. While for the production of the AMCPs, the average grain size of the AA2011-T6/nano Al_2_O_3_ composite (Figure 12g) and AA2011-O/nano Al_2_O_3_ composite (Figure 12h) were 2.07 and 1.75 µm, respectively. It can be concluded that the FSD achieved a reduction in the grain size of 81.31 and 83.79% for the deposited material of AA2011-T6 and AA2011-O, respectively. Furthermore, a slight grain size refining was observed for the produced composites with the addition of nano Al_2_O_3_. While a slight reduction was detected for the addition of Al_2_O_3_ nano compared with the deposited matrices at the two temper conditions, the grain size reduction for the composites were 3.63 and 4.44% for AA2011-T6/Al_2_O_3_, and AA2011-O/Al_2_O_3_ deposited layers, respectively. It was hypothesized that during the FSD that produced the AMCPs in the two temper conditions, the aluminum matrix material developed a significantly higher dislocation density owing to the differences in the deformation and the thermal characteristics of the matrix and the Al_2_O_3_ reinforcement nanoparticles. Consequently, during the FSD to produce AMCPs, hot restoration processes occurred more actively in the matrix material resulting in a finer grain size.

**Figure 13:**
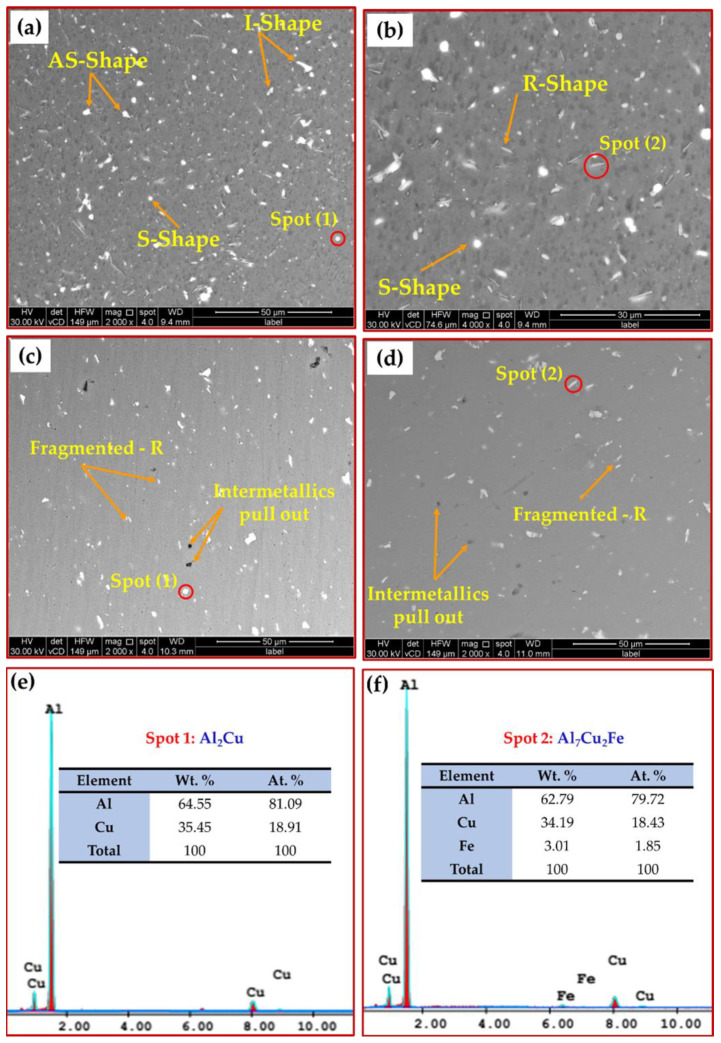
(**a**) Low and (**b**) high magnification SEM images of AA2011-T6 BM, (**c**) deposited matrix, and (**d**) AA2011/nano Al_2_O_3_ composite; (**e**,**f**) EDS analyses of spot 1 and spot 2, respectively, in (**a**–**d**).

**Figure 14:**
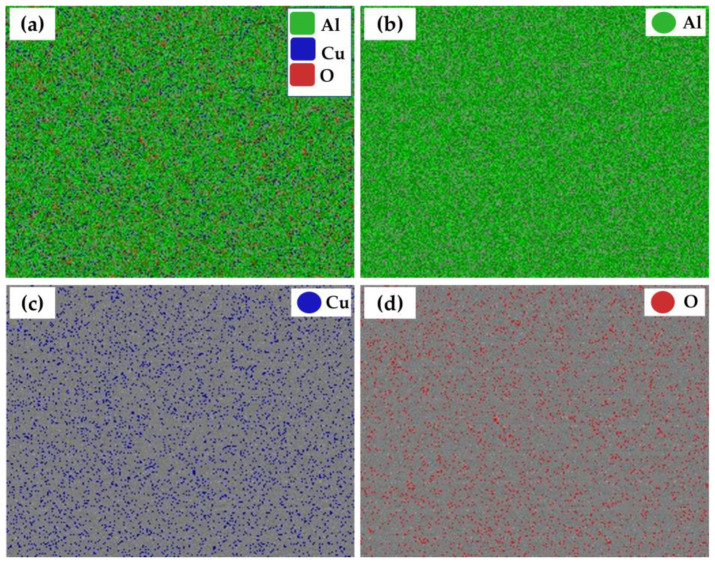
Elemental map distribution of the deposited AA2011/nano Al_2_O_3_ composite.

The intermetallic precipitates in the AA2011-T6 BM, deposited matrix, and AA2011/nano Al_2_O_3_ composite were examined using a SEM equipped with EDS analysis. The SEM analysis showed different morphologies of intermetallics: spherical (S); almost spherical (A–S); irregular (I); rod-like (R), as given in Figure 13a–d. According to the chemical composition of the AA2011-T6 (Table 1), only two intermetallics were detected: Al_2_Cu and Al_7_Cu_2_Fe [46,56]. It can be remarked that the precipitate size decreased with the stirring action during the deposition process for the deposited matrix (Figure 13c) and the deposited composite (Figure 13d) compared to the as-received AA2011 BM (Figure 13a,b). The fragmentation and dispersion of these intermetallics in the aluminum matrix has been reported in other works [42,46,57]. The first intermetallic Al_2_Cu (Spot 1), as shown in Figure 13a,c, was presented in different morphologies (i.e., S, A–S, and I shapes) through the AA2011-T6 and the deposited materials. Its nominal composition is given in Figure 13e. The second detected intermetallic was Al_7_Cu_2_Fe (Spot 2), as shown in Figure 13b,d. Its morphology was an R shape, and the nominal composition is illustrated in Figure 13f. It can also be noticed that these intermetallics were well coherent with the aluminum matrix AA2011-T6 BM (Figure 13a,b) without pulling out. During the grounding and polishing of the deposited materials, the pullout phenomena were detected (Figure 13c,d), indicating the weak interface bond between the intermetallics and the matrix after FSD. The elemental map distribution of the reinforcement nano Al_2_O_3_ phase and the high alloying element (Cu) in the AA2011 alloy are dotted in different colors (Figure 14) to ensure the dispersion of the reinforcement and the intermetallic Al_2_Cu in the aluminum matrix after FSD. Figure 14a shows the map distribution of all elements. Figure 14b–d represent the elemental maps of Al, Cu, and O, respectively.

### 3.3. The Physical and Mechanical Properties

**Figure 15:**
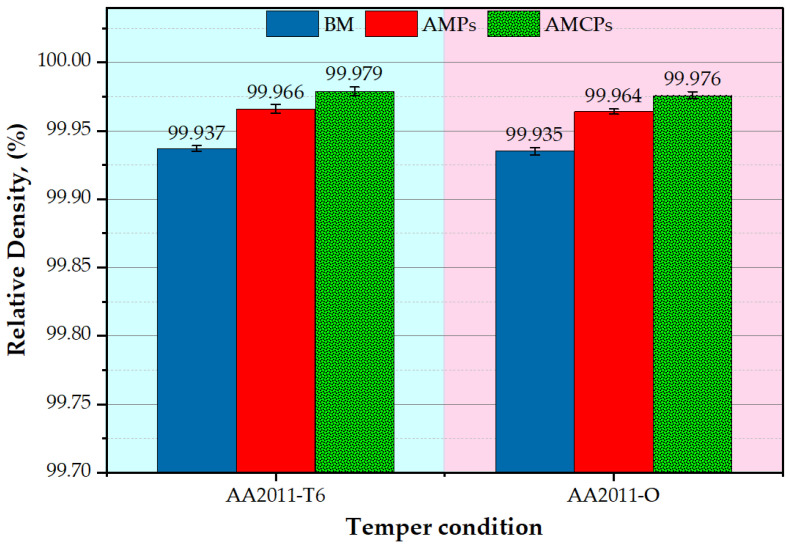
The relative density of the BM, AMPs, and AMCPs.

Figure 15 illustrates the variation in the relative density of the initial materials (i.e., AA2011-T6 and AA2011-O) and the produced AMPs and AMCPs. It can be seen that no less than a 99.935% relative density for all the measured specimens was attained. Moreover, a slight increase in density was observed for the friction stir deposited materials and composites compared to the as-received materials. This increase may be ascribed to the densification of the deposited building layer due to the downward force of the consumable rod during the feeding rate and the presence of the Al_2_O_3_ ceramic phase [58,59,60,61].

**Figure 16:**
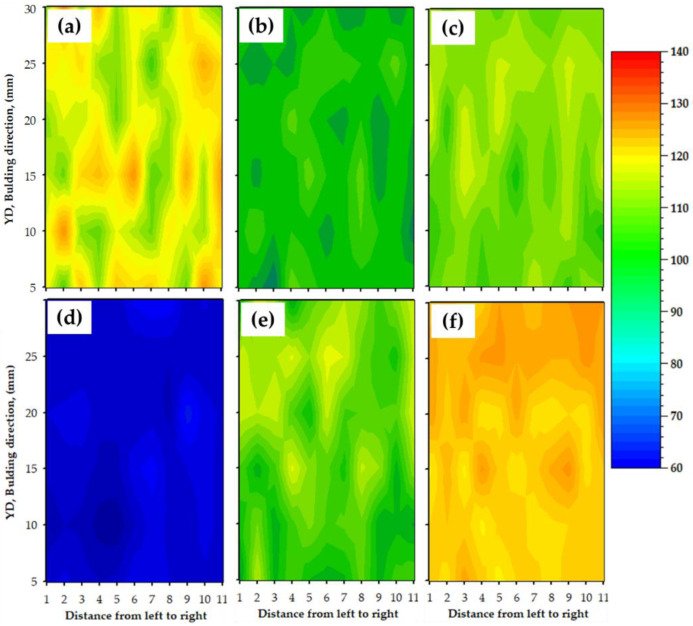
Hardness contours of the (**a**) initial material, (**b**) AMP, and (**c**) AMCP at temper condition of T6; (**d**–**f)** hardness contours for the initial material, AMP, and AMCP at the temper condition of O.

The hardness of a material is a good indicator of its mechanical characteristic, and its value is related to the initial chemical composition, heat treatment program, and deformation process parameters. The contour maps of Vickers hardness measurements of base materials, the deposited materials, and the deposited composites at a 800 rpm rotation speed and a 5 mm/min feed rate are displayed in Figure 16. These measurements through the cross-section of the tested specimens are a good representation of the hardness variation, which is related to the microstructural features and the material processing. The AA2011-T6 initial material hardness map (Figure 16a) shows a wide range of hardness measurements, ranging from 106 to 130 HV with an average hardness value of 117 HV. The high hardness of the AA2011-T6 temper condition was attributed to the high internal stresses stored in the material due to the cold working [62,63]. The drop in the hardness measurements of AA2011-O BM (Figure 16d) was attributed to the stress relief and the precipitation coarsening [64]. The hardness variation ranged from 60 to 75 HV in the annealed state with an average of 69 HV, and the wide range variation of measurements for the two temper conditions of T6 and O was likely due to the different morphologies, sizes, and distribution of the precipitates (Figure 10b and Figure 13a,b, respectively). It can also be seen that the FSD process showed more homogenous hardness distribution (narrow range of hardness measurements) across the build materials of AMPs (Figure 16b,e) and AMCPs (Figure 16c,f) compared to the initial materials, indicating an isotropy structure. After the FSD of AA2011-T6, the AMP exhibited a hardness map variation (Figure 16b) ranging from 87 to 98 HV with an average of 91 HV. At the same time, the deposited AA2011-O revealed a variation in hardness (Figure 16e) from 100 to 118 HV with an average of 107 HV. Furthermore, the hardness variation of AA2011-T6/nano Al_2_O_3_ composite ranged from 101 to 112 with an average of 106 HV, and for the AA2011-O/nano Al_2_O_3_ composite, it ranged from 116 to 125 HV with a 118 HV average hardness. It is widely established that two mechanisms regulate the hardness of this alloy. The first mechanism is the decrease in grain size, which increases with hardness (Hall–Petch relation). The second mechanism is the shape and dispersion of the precipitates. In friction stir deposited material using the AA2011-T6 hard consumable rod, the hardness decreased after the deposition process. The dominant mechanism that affected the hardness was the fragmentation of hard precipitates, while the hardness of the deposited material using the AA2011-O rods enhanced after the deposition, as the dominant mechanism is the grain size refining. The addition of nano Al_2_O_3_ to produce the AMCPs improved the hardness values for the two deposited matrices. This enhancement in hardness was ascribed to the high hardness nature of the Al_2_O_3_ nanoceramic particles with it high dispersion through the friction stir deposited materials. Moreover, the individual Al_2_O_3_ nanoparticles suppressed grain coarsening by impeding the motion of grain boundaries, resulting in grain refining [65,66]. In general, it can be said that the hardness properties of the deposited AMPs and AMCPs depended on the grain size, nanoparticles addition, temper conditions, dislocation density, and precipitates (i.e., morphology, dispersion, and size).

**Figure 17:**
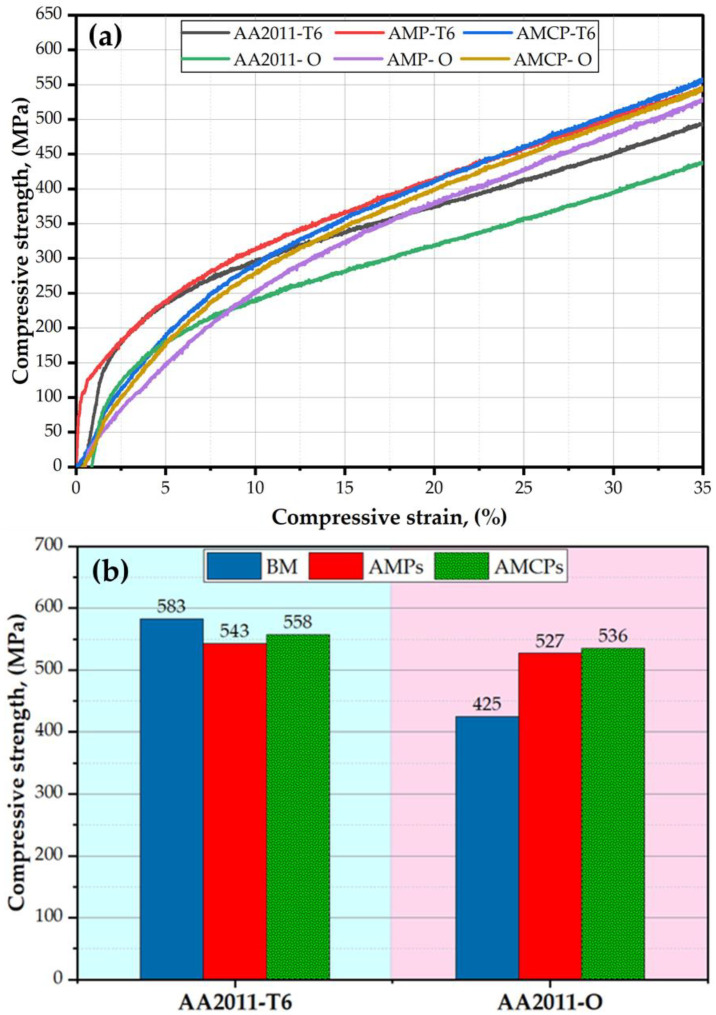
(**a**) Stress–strain compression curves of the initial materials, AMPs, and AMCPs for AA2011-T6 and AA2011-O; (**b**) compressive strength values at a strain of 35% for the initial materials, AMPs, and AMCPs at both temper conditions of T6 and O.

The compression test is critical for determining how the examined materials behave under compressive load. The stress–strain compression curves and the compressive strength at a 35% strain of the initial materials, AMPs, and AMCPs are plotted in Figure 17a,b, respectively. It can be seen that the compressive strength of the AA2011-T6 deposited material (543 MPa) was lower than the AA2011-T6 initial material (583 MPa) due to the hardness loss accompanying the exposure of the thermomechanical process during FSD [67]. The addition of Al_2_O_3_ promoted a slight enhancement in the strength of the AA2011-T6/nano Al_2_O_3_ composite (558 MPa) compared to the deposited material without Al_2_O_3_ due to the increase in thermal stability and hardness associated with the presence of the nanoceramic phase [68,69]. The compressive strength of the AA2011-O deposited material (536 MPa) and its composites (527 MPa) improved over the AA2011-O BM (425 MPa) due to the fact of grain refining and the nano Al_2_O_3_ addition.

**Figure 18:**
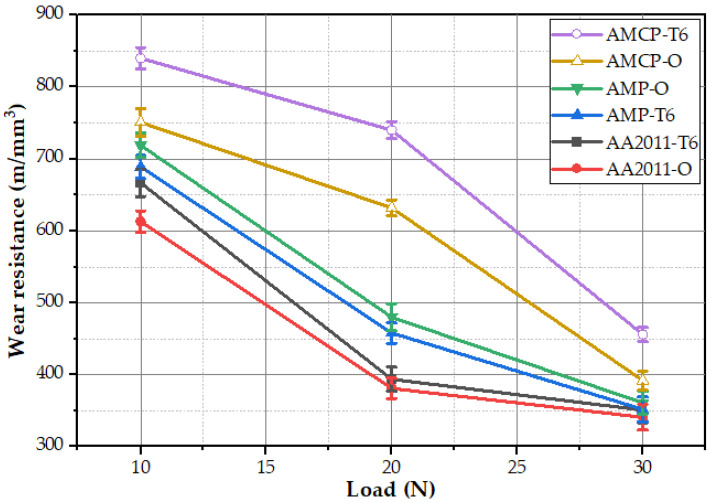
Wear resistance of the initial materials, AMPs, and AMCPs at both temper conditions.

**Figure 19:**
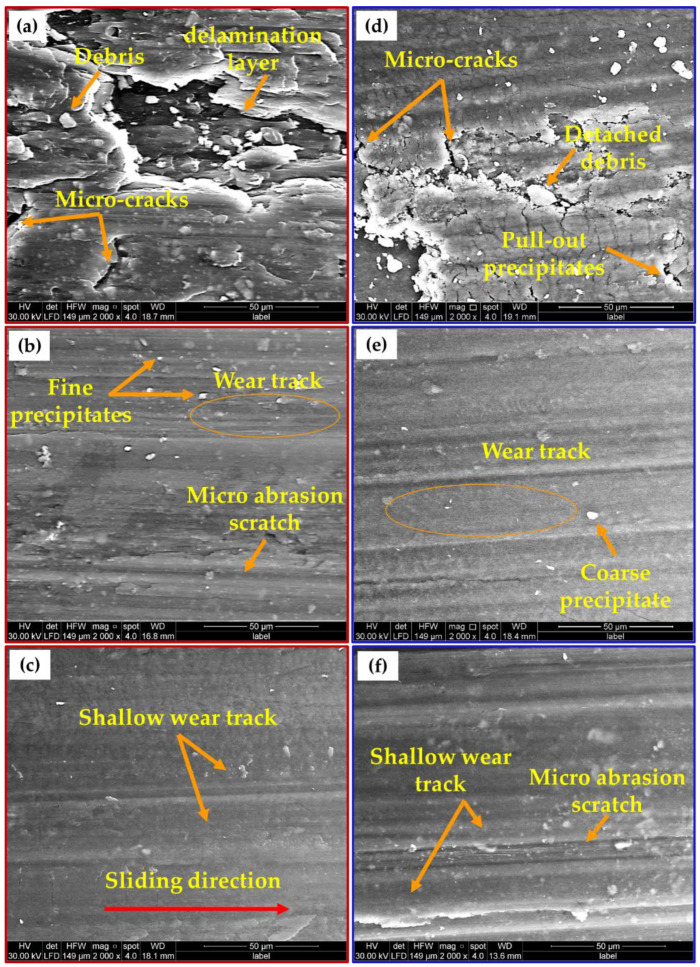
SEM images of the worn surface of the initial materials, AMPs, and AMCPs: (**a**–**c**) T6 and (**d**–**f**) O temper conditions, respectively.

Wear resistance of a material refers to its capacity to withstand material loss due to the fact of mechanical activity. Wear resistance is calculated as the inverse of the wear rate (which is the volume loss per sliding distance), and its value is related to the material type, microstructure features, and the wear test conditions. The wear behavior of the initial materials, AMPs, and AMCPs in terms of wear resistance were examined at different applied loads from 10 to 30 N at a constant sliding distance of 2072 m. The wear resistance results were plotted against load as given in Figure 18. In general, the applied loads were sufficient for surface damage, and the wear resistance of all of the tested specimens decreased with the increasing applied load. The friction stir deposited materials showed a slight wear resistance improvement over the initial two temper conditions. Moreover, both the produced composites showed higher wear resistance than the deposited material without nano Al_2_O_3_ addition and the initial materials. The AA2011-T6/nano Al_2_O_3_ composite attained the highest wear resistance at all the applied loads. These results agree well with the density, hardness, and strength results, except for the AA2011-T6 BM. Figure 19 shows SEM images of the worn surfaces of the initial materials, AMPs, and AMCPs at the wear load of 20 N and a sliding distance of 2072 m. The worn surface of the initial materials shows clear damage (Figure 19a,d) in terms of the delamination layer, transverse micro-cracks, detached debris, and small smooth regions interspersed with wear scars. Due to the rise in temperature of the AA2011 aluminum surface throughout the wear process, the applied friction force induced cyclic stresses on both sides of the rubbing surfaces. This temperature increase weakened the hardness and strength of the aluminum, resulting in significant plastic deformation and the peeling off of certain aluminum grains [70,71]. The delamination wear mechanism produced delamination layers (deep grooves), while microcracks were produced by the adhesive wear mechanism [72]. The different morphologies of the intermetallics (i.e., Al_2_Cu and Al_7_Cu_2_Fe) imparted detrimental effects on the worn surface during the dry friction process (Figure 19a). They pulled out through the process between the two rubbing surfaces (the specimen surface and the test rig surface), causing weight loss in terms of micro-abrasion scratches. Considerable wear resistance was attained for the deposited materials and their composites as shown by the features of the worn surfaces, as shown in Figure 19b,e and Figure 19c,f, respectively, in terms of parallel shallow wear tracks with the absence of microcracks and delamination layers. Moreover, micro-abrasion scratches were detected on the worn surface due to the loose fragmented intermetallics between the two rubbing surfaces (Figure 19b,e). The enhancement in wear resistance of the deposited composites was due to the increase in material thermal stability with nano Al_2_O_3_ ceramic addition accompanied by hardness improvement due to the grain refining.

## 4. Conclusions

1. The FSD process successfully deposited sound continuous multilayers of the AA2011 at the two temper conditions of T6 and O with and without the addition of Al_2_O_3_ nanoceramic particles at a rotation speed of 800 rpm and a feed rate of 5 mm/min;
2. The hard material AA2011-T6 consumable rod showed stronger resistance and took a longer time to deposit, achieving higher building material than the soft material AA2011-O rod during the FSD at a constant processing parameter;
3. The FSD process showed a more homogenous hardness distribution with a narrow range of hardness measurements across the built materials of AMPs and AMCPs than the base materials;
4. The compressive strength of the deposited composites was higher than the deposited matrices without Al_2_O_3_ in the two temper conditions;
5. The deposited composites showed higher wear resistance than the deposited AA2011 matrices and the base materials at a sliding distance of 2072 m with the applied load ranging from 10 to 30 N. Furthermore, the AA2011-T6/nano Al_2_O_3_ composite showed the highest wear resistance among all the deposited and base materials.

## Figures and Tables

**Table 1:** Chemical composition of the initial AA2011 material.

Elements	Cu	Si	Fe	Ti	Bi	Zn	Pb	Ni	Al
Wt.%	5.12	0.39	0.70	0.31	0.22	0.24	0.20	0.04	Balance

**Table 2:** Dimensions and standards of the cut specimens for different tests.

Specimen No.	Deposited Material (mm)	Test	Standard
(a)	∅10 × 32	Compression	ASTM E9
(b)	∅10 × 32	Wear	ASTM G99
(c)	10 ×10 × 32	Density	JIS R2205-1992
(d)	25 × 10 × 32	Hardness and microstructure	ASTM E92

## Data Availability

Data will be available upon request through the corresponding author.

## Abbreviations

Al-MCCs	Aluminum matrix ceramic composites
PM	Powder metallurgy
FB-AM	Fusion-based additive manufacturing
SS-AM	Solid-state based additive manufacturing
FAM	Friction additive manufacturing
FSD	Friction stir deposition
AMCs	Additive manufacturing composites
AMPs	Additive manufacturing parts
AMCPs	Additive manufacturing composite parts
FSDed	Friction stir deposited
FSW	Friction stir welding
FSP	Friction stir processing
BM	Base material
DCF	Dynamic contact friction
SPD	Severe plastic deformation
SZ	Stir zone
S shape	Spherical shape
A–S shape	Almost spherical shape
I shape	Irregular shape
R shape	Rod-like shape
